# Supplementary material for: Structure and pigment make the eyed elater’s eyespots black
Source: PeerJ. 2020 Jan 13;8:e8161. doi: 10.7717/peerj.8161 (PMC6964691; doi:10.7717/peerj.8161)
Supplement: Supplemental Information 1 — Figure S1, graphical denotation of the rotation of the setae within the eyespot. Supplementary Figure S2, energy dispersive X-ray spectrometry (EDS) sample areas. Figure S3, EDS spectra of sample areas. Figure S4, scanning electron micrograph of the bases of eyespot setae that shows magnified view (25,000×magnification) without building of charge indicating thorough sputter coating of platinum and palladium metals. [file peerj-08-8161-s001.docx]

**Supplementary material**

**Distribution of setae within the eyespot**

**
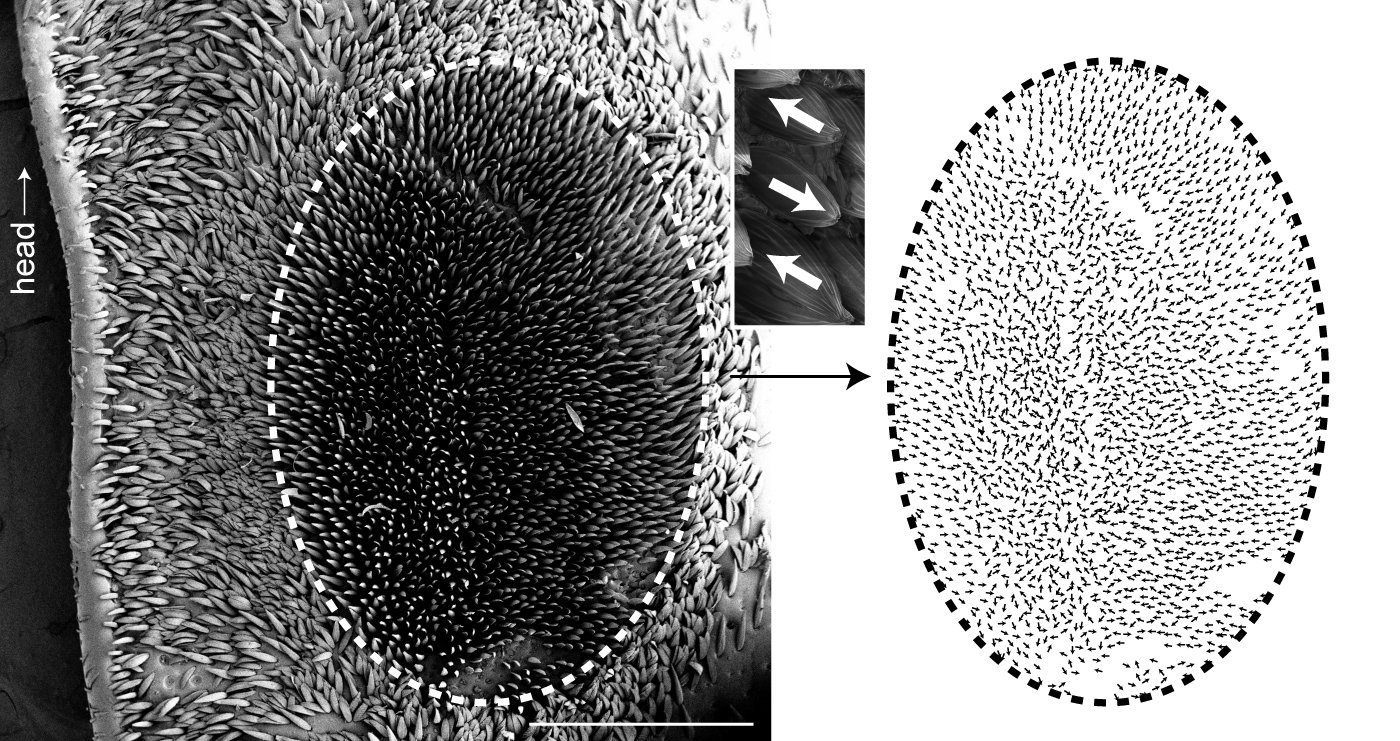
Supplementary Figure 1**: Graphical denotation of the rotation of the setae within the eyespot, shown by arrows. Inset: the point of the arrow is on the convex side of the seta, and the nock of the arrow is on the convex side. Scale bar = 1 mm

**Energy dispersive X-ray spectrometry (EDS)**


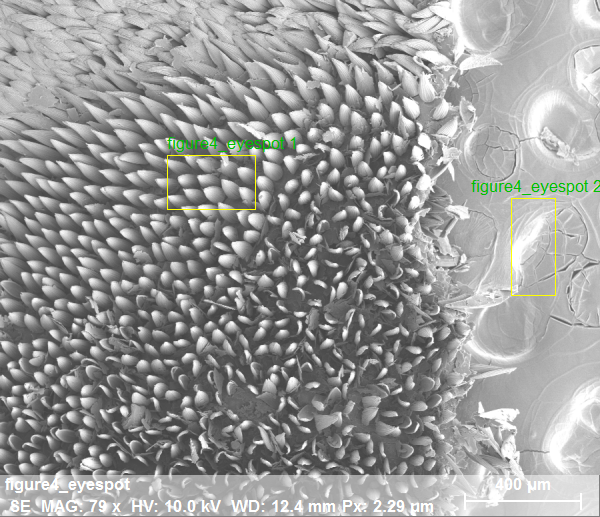


**Supplementary Figure 2**: The image to the left shows the two sample areas (framed in yellow boxes) of the EDS analysis: (1) palladium (Pd) and platinum (Pt) coated setae within the eyespot (red text), and (2) Pd-Pt coated stub surface (green text). EDS settings were as follows: Magnification:79.48X, HV:10.0kV, Date: 8/13/2019, 4:30:31, image name: PMImage size: 900 x 77 pixels

Area 1: eyespot setae

Area 2: stub surface

Energy (keV)

Area 1: eyespot setae

Area 2: stub surface

X-ray counts (cps)

**Supplementary Figure 3**: The image above shows EDS spectra of the two sample areas: (1) coated setae within the eyespot (red text) and (2) coated stub surface (green text). X-ray counts (cps) are on the Y-axis and energy (keV) is on the X-axis The red and green spectra have the same profile indicating identical elemental composition and uniform coating. EDS settings were as follows: (1) eyespot sample area with Puls th.: 3.37 kcps, (2) stub surface with Puls th.: 7.05 kcps.

**Scanning electron microscopy**


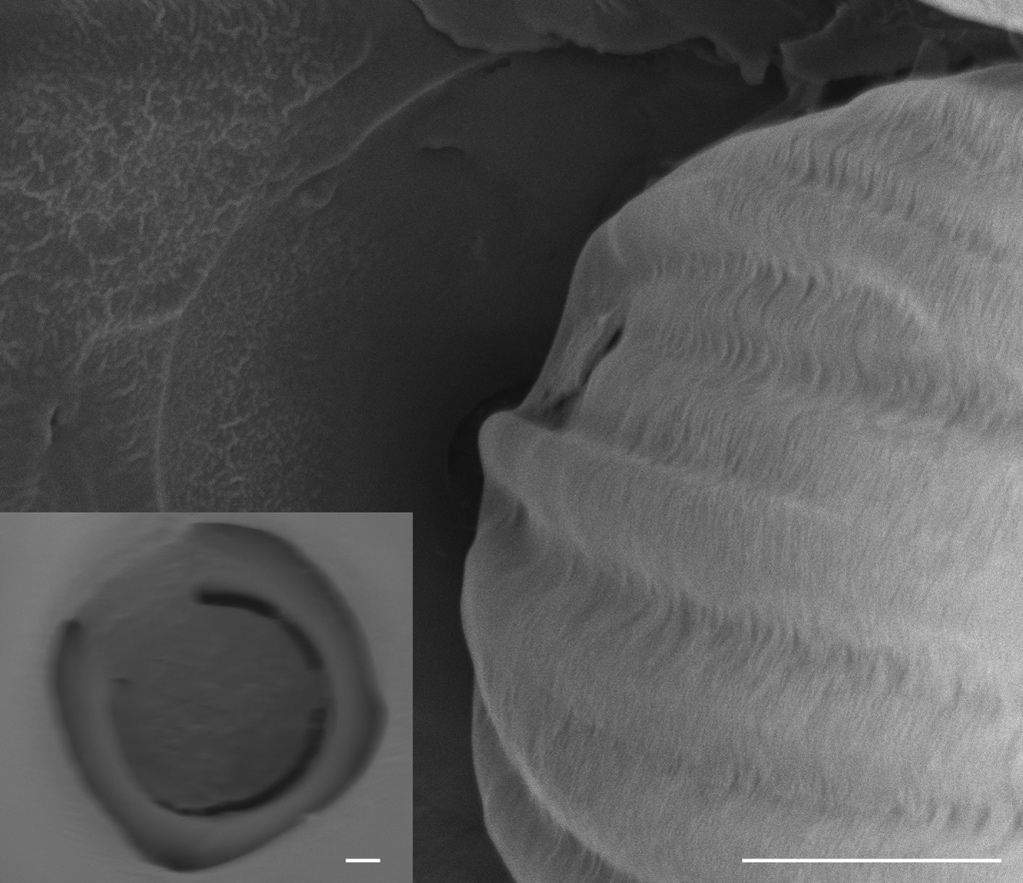


**Supplementary Figure 4**: The images above are scanning electron micrographs of the bases of eyespot setae. The inset image is of the setal socket with the seta removed. The image shows that high magnification is easily achieved without building of charge. The scale bars = 10 um and 800 nm for the inset.
